# Successful recovery following musculoskeletal trauma: protocol for a qualitative study of patients’ and physiotherapists’ perceptions

**DOI:** 10.1186/s12891-021-04035-9

**Published:** 2021-02-10

**Authors:** N. Middlebrook, N. R. Heneghan, D. Falla, L. Silvester, A. B. Rushton, A. A. Soundy

**Affiliations:** 1 Centre of Precision Rehabilitation for Spinal Pain, School of Sport, Exercise and Rehabilitation Sciences, College of Life and Environmental Sciences, University of Birmingham, Edgbaston, Birmingham, B15 2TT UK; 2 University Hospitals Coventry and Warwickshire NHS Trust, University Hospital, Clifford Bridge Road, Coventry, CV2 2DX UK; 3 Western University, School of Physical Therapy, London, Ontario N6G 1H1 Canada; 4 School of Sport, Exercise and Rehabilitation Sciences, College of Life and Environmental Sciences, University of Birmingham, Edgbaston, Birmingham, B15 2TT UK

**Keywords:** Musculoskeletal trauma, Recovery, Semi-structured interviews, Focus groups

## Abstract

**Background:**

Annually in the UK, 40,000–90,000 people are involved in a traumatic incident. Severity of injury and how well people recover from their injuries varies, with physiotherapy playing a key role in the rehabilitation process. Recovery is evaluated using multiple outcome measures for perceived levels of pain severity and quality of life. It is unclear however, what constitutes a successful recovery from injury throughout the course of recovery from the patient perspective, and whether this aligns with physiotherapists’ perspectives.

**Methods:**

A qualitative study using two approaches: Interpretive Phenomenological Analysis (IPA) using semi-structured interviews and thematic analysis following the Kreuger framework for focus groups. A purposive sample of 20 patients who have experienced musculoskeletal trauma within the past 4 weeks and 12 physiotherapists who manage this patient population will be recruited from a single trauma centre in the UK. Semi-structured interviews with patients at 4 weeks, 6 and 12 months following injury, and 2 focus groups with physiotherapists will be undertaken at one time point. Views and perceptions on the definition of recovery and what constitutes a successful recovery will be explored using both methods, with a focus on the lived experience and patient journey following musculoskeletal trauma, and how this changes through the process of recovery. Data from both the semi-structured interviews and focus groups will be analysed separately and then integrated and synthesised into key themes ensuring similarities and differences are identified. Strategies to ensure trustworthiness e.g., reflexivity will be employed.

**Discussion:**

Recovery following musculoskeletal trauma is complex and understanding of the concept of successful recovery and how this changes over time following an injury is largely unknown. It is imperative to understand the patient perspective and whether these perceptions align with current views of physiotherapists. A greater understanding of recovery following musculoskeletal trauma has potential to change clinical care, optimise patient centred care and improve efficiency and clinical decision making during rehabilitation. This in turn can contribute to improved clinical effectiveness, patient outcome and patient satisfaction with potential service and economic cost savings. This study has ethical approval (IRAS 287781/REC 20/PR/0712).

**Supplementary Information:**

The online version contains supplementary material available at 10.1186/s12891-021-04035-9.

## Background

Traumatic injury is common, with an estimated 973 million people sustaining injuries which warranted healthcare input [1]. Annually in the UK, 40,000–90,000 people are involved in a traumatic accident [2–4], with approximately 50% resulting in musculoskeletal injury [2]. The two most common mechanisms of musculoskeletal injury are road traffic accidents and falls from less than 2 m [4, 5]. The severity of injury can vary significantly but with advances in healthcare, more people are surviving their injuries [1]. The financial burden of traumatic injuries in the UK is significant, with the National Audit Office estimating that in 2010, the lost economic output as a result of trauma was £3.3 billion [3].

Rehabilitation is recognised by the Trauma and Audit Research Network (TARN), and the National Health Service (NHS) of the UK as a high priority during the recovery process, with national guidelines recommending that patients with more severe injuries are routinely referred to physiotherapy [6]. It is well documented that recovery can be a long process, often beyond 12 months [7]. Previous studies have reported persistent pain, poor rates of return to work and psychological manifestations such as anxiety as indicators of poor recovery [7, 8].

Recovery has been described from qualitative studies with mild musculoskeletal injury as a complete resolution of symptoms e.g. pain, and restoration of function without symptoms, or recovery of function with residual symptoms [9]. Recovery following musculoskeletal trauma is often quantified using outcome measures related to domains such as pain (e.g. pain rating scales), functional outcomes evaluating activities of daily living (e.g. SF-36) [10], or survival rates after 30 days – the main outcome used by TARN [11]. There is however limited research around the topic of recovery and what is deemed ‘successful’ following musculoskeletal trauma, with studies focusing on patient experience of the trauma service rather than their perception of recovery [12].

Two studies have explored recovery following major musculoskeletal trauma [13, 14], with one study interviewing patients at 3–6 months [13] and the other at 3, 4 and 5 years following injury [14]. Through semi-structured interviews, both studies highlighted the complexity of establishing a definition of recovery, not least because perceptions of recovery change over time [13]. Findings also demonstrate how multiple factors such as pain, disability and return to work are impacted by anxiety and uncertainty following injury [14]. The themes of pain and disability are echoed in previous qualitative studies specifically focused to open lower limb fractures [15–17]. However, themes around a sense of vulnerability and strong emotional feelings were identified as contributing to the concept of recovery at both the acute [16] and long term stage following injury [17]. Nevertheless, views around recovery differed between patient and clinicians which included both physiotherapists and other allied health professionals and orthopaedic surgeons. Themes around psychological and financial impacts were deemed important in recovery following ankle fractures for patients but not clinicians [18], highlighting the importance of evaluating the concept of recovery from a patient and clinicians perspective.

No study has evaluated both patients’ and physiotherapists’ perspectives of what constitutes a successful recovery or has followed the ‘patient journey’ from onset and throughout recovery to evaluate how perceptions of recovery could change over time. Through semi-structured interviews with patients who have sustained a traumatic injury to explore their views of recovery, interviews will be conducted at 4 weeks, 6 and 12-months post-injury. In addition, we will conduct focus groups to explore physiotherapists’ with varying levels of experience views of recovery. Understanding perceptions of recovery from onset and throughout the recovery process in combination with physiotherapist perceptions are important in order for future research focused to recovery in musculoskeletal trauma to be directed and informed by patients and physiotherapists which is relevant and beneficial for them.

### Aims and objectives

The aim of this study is to explore patients’ and physiotherapists’ views and perceptions of the definition of recovery, and of what constitutes successful recovery from the point of injury to later stages of recovery following musculoskeletal trauma. The objectives are:

1. To understand the patient journey following musculoskeletal trauma and whether perception of recovery changes through early, mid to late stages of recovery (4 weeks, 6 and 12 months).
2. To explore patients’ views and perceptions on the definition of recovery and if/when they perceive they have achieved a successful recovery.
3. To explore the physiotherapists’ perceptions of what they define as a successful patient recovery.
4. To explore views and perceptions of physiotherapists regarding outcome measures that are useful to assess recovery.

## Methods and design

### Design and theoretical framework

This qualitative study using semi-structured interviews and focus groups has been designed using the consolidated criteria for reporting qualitative research (COREQ) [19], and informed by the study management team. A qualitative study using two approaches will be employed: - Interpretive Phenomenological Analysis (IPA) using semi-structured interviews and thematic analysis following the Kreuger framework for focus groups.

IPA will be undertaken to explore the patient experience and perceptions of recovery following musculoskeletal trauma. IPA is an approach that allows for an exploration of the experience of an event from an individual’s perspective. The approach seeks to explore how individual participants make sense of the event and also to understand the meaning behind these experiences [20], making this method appropriate to explore the concept of recovery following musculoskeletal trauma.

A focus group allows group interaction and discussion around the topic of recovery and their perceptions, whereby group interactions can be observed as well as gaining insight into the knowledge, attitudes and experiences of the participants [21, 22]. For the focus groups, a thematic analysis is more appropriate form of analysis as the focus group seeks to explore views, perceptions and attitudes to recovery but not in depth into a particular experience making a thematic analysis following the Kreuger Framework more appropriate [23]. Data from both the interviews and the focus groups will be analysed separately in the first instance and then synthesised to gain an overall understanding around recovery comparing and contrasting physiotherapist and patient perceptions.

**Fig. 1:**
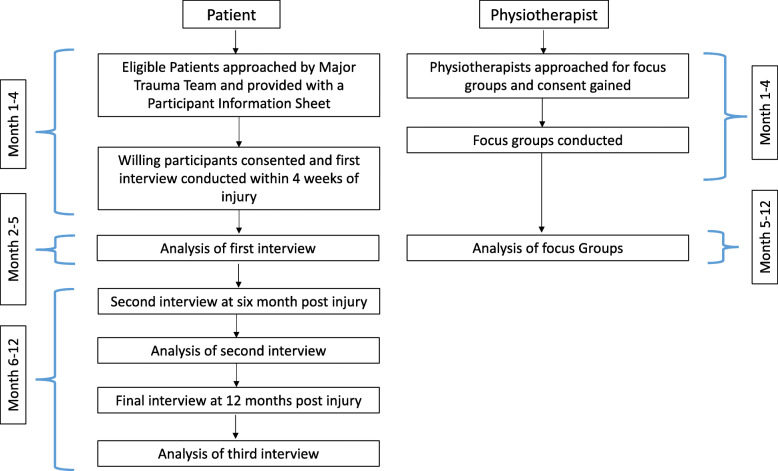
Study flow diagram

The first interviews and focus groups will be conducted concurrently in order to prevent any bias from the researcher i.e. further exploration based on responses from patients in the focus groups and vice versa. An overview of the study is summarised in Fig. 1.

### Study setting

One UK major trauma centre - University Hospital, University Hospitals Coventry and Warwickshire NHS Trust.

## Methods

### Patient interviews

Participants will be invited to complete 3 semi-structured interviews. The interviews will be scheduled at time points to capture any changing views and perceptions the participants may experience through the course of recovery. The first interview will be completed within 4 weeks of injury. The second and third interviews will be conducted at 6 and 12-months following injury. These time points enable mapping of the course of recovery over time from early to later stage of injury [8, 24]. Following informed consent, interviews will be conducted at the convenience of the patient. Due to the traumatic nature of the injuries, participants will have the option of ‘non-participants’ e.g. family member, to be present for the duration of the interview.

Due to COVID-19, various actions have been taken to ensure the safety of both patient and researcher. These actions are summarised in Table 1.

**Table 1:** Summary of measures taken due to COVID-19 to ensure data collection can continue over 12 months

Potential disruption or risk	Actions taken
Face to face interviews cannot take place	1. To minimise risk to patient and researcher the first interview will be conducted via a video call (Microsoft team) or alternatively a telephone call will be used if the patient does not have access to video calling.
Minimising risk of COVID-19 transmission for second and third interviews	1. Second and third interviews can be conducted via video call (Microsoft teams) or telephone call if participant cannot access video calling. 2. Face to face interviews can take place when safe to do so in either the University setting or at patients home; if and when current government guidance changes and in line with University policy.
Recording of interviews if conducted via video call or telephone	All interviews will be audio recorded using the same password protected digital recorder.

A topic guide for interviews (supplementary documents 1–3) has been developed by the research team including our patient co-investigator, using knowledge from a current study [24]; and relevant systematic reviews [25]. The topic guide is informed by the International Classification of Function, Disability and Health (ICF) domains of body functions, body structure, activity and participation and environmental factors [26]. The topic guide for the first interview will explore the patient’s current injury/injuries and function, their description of recovery at < 4 weeks following injury and what they define as a successful recovery at this stage of their recovery process (objectives 1 & 2). The second and third interview will follow a similar structure, with data analyses at an individual level from the first interview informing adaptation/development of questions in subsequent interviews. All interviews will be conducted by one researcher (NM). Training will be acquired from experienced researchers with expertise in IPA approach (AS, AR) to ensure quality. To ensure feasibility of the topic guides, pilot interviews will be conducted with our patient co-applicant, with a further two pilot cognitive interviews conducted with patients who have previously experienced musculoskeletal trauma (members of the Centre of Precision Rehabilitation for Spinal Pain (CPR Spine) spinal register/PPI group).

All interviews are voluntary with participants free to stop at any time. If a participant becomes distressed during the interview in accordance with our risk assessment for the study, appropriate action by the interviewer, a registered healthcare practitioner, will be taken to stop the interview and provide further support for the patient, if required (e.g. signposting to services such as Forward Thinking and Mind) [27]. All interviews will be audio recorded and transcribed verbatim and data will remain confidential at all times in line with Good Clinical Practice (GCP) processes. Through the process of ‘member checking’, interview transcriptions will be returned to participants to review and allow the participant the ability to reflect on their account and give further insight and make additions as required [28].

Loss to follow up is common in this population [24], therefore, every effort by the Chief Investigator (CI) (NM) will be made during and after the first interview process to build rapport and trust with patients ensuring that they are comfortable with the study processes. At least two methods of contact will be documented (personal email and phone number- home and mobile) and contact details for next of kin. Contact for subsequent interviews will be made by either email or telephone at the participant’s preference 2–3 weeks before the interview to schedule an interview date and time at the participants convenience. A follow up email or phone call will take place 1 week later if no contact is established. If required and unable to contact the patient by their preferred method e.g. telephone, the alternative method of email or next of kin details will be used. A maximum of 3 attempts to contact the patient will be used.

### Physiotherapist focus groups

The focus groups will be led by NM with assistance from co-applicants who are experienced in conducting focus groups (AR, NH). Due to COVID-19 and to ensure government and local policy on social distancing are adhered to, the focus groups will be conducted virtually with video calling using Microsoft Teams which is the Trust approved software and accessible to all Trust physiotherapists. The focus groups will be audio recorded using a password protected digital recorder.

A topic guide has been developed by the research team (supplementary document 4), and developed in line with the patient semi-structured interview topic guides to ensure similar topics are covered. Questions will focus on physiotherapists’ views and definitions of a successful recovery (objective 3) and views around outcome measures currently used to assess recovery (objective 4).

An experienced moderator (AR or NH) will be present for the focus groups and will monitor progress and take field notes. Prior to the focus groups, the topic guide will be discussed with the moderator as part of training for NM. As a wide range of physiotherapist experience levels will be present in the focus groups, in order to ensure all participants feel comfortable in expressing their views during the focus group, it will be stated at the beginning of the group that every opinion matters and we are interested in everyone’s personal experience and welcome all opinions as well as emphasising that the focus group content will be kept confidential.

### Participants

#### Patient interviews

A purposive sample (approximately *n* = 20) will be recruited from one NHS major trauma centre. IPA traditionally uses small sample sizes in order to achieve rich high quality detailed interpretative accounts with a homogenous population sample [27], although past studies have successfully employed IPA with up to 48 participants [29]. Although the musculoskeletal trauma population has heterogeneity e.g. type of injury, severity, location and structure injured, it is anticipated based on previous research that similar findings across different subgroups will be common with smaller differences depending on type and injury severity [13–17]. Therefore, the higher sample size in this study will allow for the heterogeneity of the musculoskeletal trauma population and ensuring the breadth of this population is represented, as well as taking into account potential drop outs, acknowledging that loss to follow up is common in this population [24].

#### Focus groups

A purposive sample of physiotherapists (approximately *n* = 10–12) will be recruited. The sample size allows for a range of inpatient and outpatient physiotherapists as well as levels of experience to give a broad representation of views of those involved in rehabilitation across the recovery period. A focus group will allow discussion to be generated around the topic with a range of musculoskeletal trauma physiotherapists. We anticipate that 2 focus groups with a total of 10–12 physiotherapists will be sufficient for data saturation in terms of the aims and objectives of this study [30], and will be reviewed following preliminary data analysis to ensure no additional themes are identified.

### Eligibility criteria

#### Patient interviews

##### Inclusion criteria

Adults (> 18 years), who have sustained a musculoskeletal injury from a traumatic event and admitted to the major trauma/orthopaedic ward within 4 weeks of injury, have mental capacity in order to give consent (score of more than 6 on the abbreviated mental test) [31], and able to communicate in English will be eligible. Musculoskeletal injury is defined for the purpose of this study as an injury to a musculoskeletal structure e.g. bones, joints, ligaments, tendons and muscles that surround these structures – a definition used in previous studies and reviews [32, 33]. Examples of musculoskeletal traumatic injuries include road traffic accidents, falls, recreational activities e.g. skiing or sporting injuries and occupational or work related injuries [33]. In order to be inclusive of participants who have sustained major injuries and who could initially be critically ill or require surgery, a recruitment window of 4 weeks will be employed, however wherever possible recruitment and initial interview will be conducted within 2 weeks of injury.

##### Exclusion criteria

Participants who sustain an injury from a non-traumatic event or where primary injury is traumatic brain injury, spinal cord injury or a neurological injury [24].

#### Focus groups

Any qualified physiotherapist who is involved in the management of musculoskeletal trauma patients’ in the Trust will be invited to participate. This includes both inpatient and outpatient physiotherapists to capture early to later stage of recovery during rehabilitation.

### Sample identification and consent

#### Patient interviews

Potentially eligible participants will be identified via the admissions lists to the major trauma and orthopaedic wards by either by a team of independent trust research nurses or the major trauma team. Any participants interested in participating in the study will be given a participant information sheet (PIS). Recruitment from the wards allows for recruitment of a range of severity of injuries including major trauma. Sampling criteria will aim to include a range of patient characteristics including age, gender and ethnicity as well as injury characteristics e.g. single vs multiple injuries, upper and lower limb and abdominal injuries (e.g. ribs fractures) and fractures vs soft tissue injuries. As IPA aims to develop a full and interesting understanding of the data rather in contrast to other theories such as grounded theory which aims to collect data until no new themes have emerged [29], recruitment will continue until the researchers and members of the steering group feel that a rich insight around the concept of recovery has been achieved and further recruitment will not add any further understanding to the topic.

### Focus groups

Physiotherapists will be recruited from the same major trauma centre in which the patient group is recruited. Physiotherapists will be identified by the team leads for both inpatient and outpatient teams and invited to participate in the focus group, and at this stage given a PIS if interested in participating. The purposive sampling technique will include a range of experience of physiotherapists (e.g. junior to senior physiotherapists) as well as both inpatient and outpatient experience. The two focus groups will combine both outpatient and inpatient physiotherapists in each group.

### Consent

### Patient interviews

The (CI), who has GCP training, and previous experience in recruiting and consenting participants following trauma within an NHS setting, will then approach the potentially eligible participants the following day to allow adequate time for the participant to read the PIS, ask questions and gain signed consent from willing participants. Consenting participants will then be scheduled for the initial interview. In the event the CI is unable to access the hospital due to COVID-19 restrictions, a member of the major trauma team/research nurses will consent willing participants prior to the interview taking place virtually.

### Focus groups

Before the focus group commences, the CI will give an opportunity for participants to review the PIS again in order to review the aims and objectives of the study, allowing any questions potential participants may have. The consent form will be explained, and the participants will be given time to read and signed prior to the focus group taking place.

### Data analysis

As stated previously, different approaches will be taken to data analysis for the patient semi-structured interviews and focus groups which are appropriate to interviews (IPA) and focus groups (Kreuger Framework). The semi-structured interviews and focus groups will be analysed separately and then synthesised across the semi-structured interviews and focus groups to gain an overall understanding around recovery comparing and contrasting physiotherapist and patient perceptions allowing a deeper understanding of recovery.

### Patient interviews

**Fig. 2:**
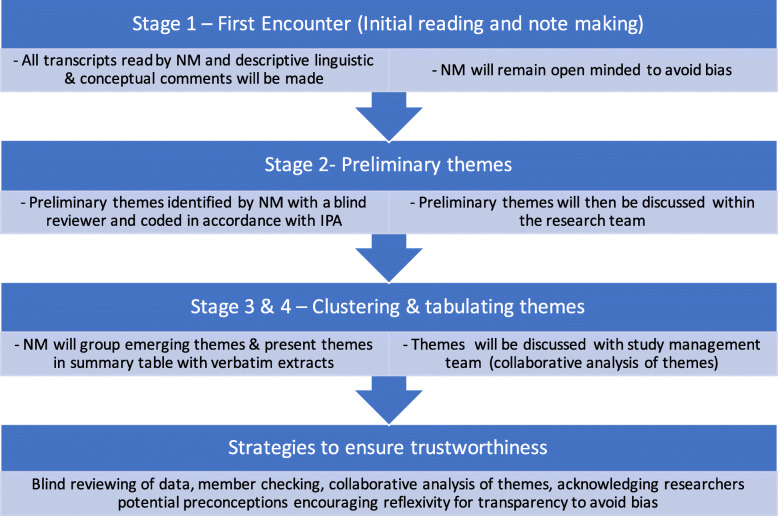
**Flow diagram summarising data analysis of patient interviews in line with IPA approach** [19, 20, 27, 28]

Data analyses will be led by the lead researcher (NM), and will take a 4-stage approach to IPA as summarised in Fig. 2.

### Physiotherapist focus groups

**Fig. 3:**
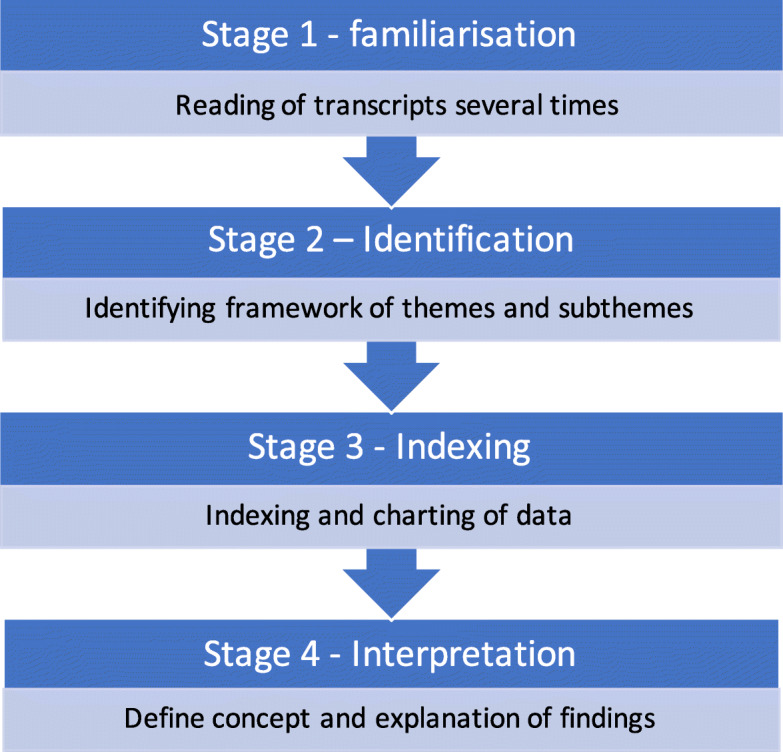
**Flow diagram summarising data analysis for focus groups in line with the Kreuger Framework** [34]

Led by the CI, four stages in line with the Kreuger Framework will be followed in the analysis process and are summarised in Fig. 3.

### Synthesis of data – semi-structured interviews and focus groups

Synthesis of the data will be conducted when the data analysis of the semi-structured interviews and focus groups has been completed. NM will look for comparing and contrasting themes around recovery and present to the Study Steering Group (SSG) for discussion. It is acknowledged combining focus group and semi-structured interviews can be challenging to ensure all relevant themes from both the interviews and focus groups are represented, therefore, the interviews and focus groups will be analysed separately as indicated above and results will then be synthesised into key themes, ensuring similarities and differences between the two groups are identified [35].

### Strategies to ensure trustworthiness

Throughout data analysis, various strategies will be employed to ensure data quality and these are summarised in Fig. 2. These include blind reviewing of the data, member checking, collaborative analysis of themes and acknowledging researchers potential preconceptions encouraging reflexivity for transparency to avoid bias [28].

### Clinical implications

Recovery following musculoskeletal trauma is complex with understanding of the concept of successful recovery and how it changes over the course of time being largely unknown. It is imperative to understand the patients’ perspectives of recovery, and if their perceptions align with the perceptions of physiotherapists. A greater understanding of recovery following musculoskeletal trauma has potential to change clinical care, increase patient centred care and improve efficiency and clinical decision-making during rehabilitation. This in turn can result in improved clinical effectiveness, patient outcome, patient satisfaction with potential service and economic cost savings. Furthermore, this study’s findings and greater understanding around the concept of recovery within this population can be a step in developing a new outcome measure to capture recovery specific to musculoskeletal trauma.

### Research governance

The study management group (SMG) which comprises the study co-applicants and principal investigator (LS), CI and an independent chair and will meet regularly throughout the duration of the study to monitor progress. The established SSG will meet regularly and will provide an overview of the study and its progression, and analyses to inform data interpretation. The SSG will include physiotherapists and academics from the University of Birmingham who have expertise in musculoskeletal trauma and qualitative research. An independent chair will provide an overview to the study. A key member of both the SMG and SSG is a person who has experienced musculoskeletal trauma and represents public involvement.

### Ethical considerations

All investigators and study site staff will comply with the GCP standards and the requirements of the General Data Protection Regulation (GDPR) and the Data Protection Act 2018 with regards to the collection, storage and processing and disclosure of personal information and will uphold the Act’s core principles.

Secure maintenance of data will ensure that any personal information collected for the purpose of follow up will be stored electronically on a password-protected computer. Data will be coded and depersonalised using a participant identification number. The linking code will be stored in a separate location on encrypted digital files. Access to these folders will be limited to the CI and AS for quality control, audit and analysis. The confidentiality of data will be preserved when data are transmitted to sponsors and co-investigators in the depersonalised format to ensure no data is traceable to any individual participant. Data will be stored for 10 years in line with the University of Birmingham’s Research Governance procedures. The data custodian will be the CI.

#### Patient interviews

There are minimal risks associated with this study for the patient interviews. Following a risk assessment, various strategies have been put in place to ensure patient privacy is maintained during interviews to ensure the participant feels comfortable to speak freely during the interview. Additionally, whilst undertaking interviews, the researcher may obtain information in which there is a concern about the wellbeing of the participant. In this situation, processes to safeguard the participant will be implemented according to local safeguarding procedures with permission from the participant to the major trauma team in the first instance to mitigate harm to the participant. Finally, due to the nature of injuries within this study, there is a potential risk of participants becoming distressed during the interview. Participants will be informed prior to starting interviews that all interviews are voluntary and that they are free to stop the interview at any time, and all participants will be reminded that they do not have to answer a particular question if do not wish to. If a participant wishes to withdraw or is lost to follow up from the study, data will be used up until the point of withdrawal. All interview recordings will be made confidential so no patient information can be identified during transcription.

#### Physiotherapist focus groups

Following a risk assessment, there are minimal risks for the focus groups. The risk of coercion from mangers/senior members of staff into participating in the focus groups will be minimised by information in participants information sheet stating the focus groups are voluntary and if individuals do not wish to participate this will be kept confidential. Participants will be informed that they can withdraw from the study at any time prior to the focus group taking place. After the focus group has been completed, the participant will have 4 weeks after the focus group has taken place to withdraw before data analysis commences. Focus group recordings will follow the same procedure as the interview transcriptions.

### Patient and public involvement

Patient and Public Involvement (PPI) has been integral to this research study from inception. The original idea for this project arose following feedback and discussion around the concept of recovery and what this means following a PPI event. Our research group at CPR Spine has established PPI input and advisors. Our PPI co-applicant has been part of the study from inception and will continue throughout the lifespan of this project. This includes feedback at the time of protocol development and topic guides and continued participation in the SSG.

### Planned dissemination

Dissemination activities will target professional, patient, and public domains at a national and international level. Professional dissemination includes submission of a manuscript of the findings of the study upon completion, and submission of abstracts to national and international conferences. Patient and public dissemination includes a lay summary, which will be available to all participants upon request and to our established PPI group members, as well as presentation of findings at the PPI group meetings. The lay summary will be made available to various trauma support groups.

### Peer review

This study has been independently peer reviewed to support funding by the Chartered Society of Physiotherapy, specifically the Physiotherapy Research Foundation Scientific Committee. This has allowed independent expert peer review.

## Supplementary Information

**Additional file 1.** Topic Guide – 1st Patient Interview.

**Additional file 2.** Topic Guide – 2nd Patient Interview.

**Additional file 3.** Topic Guide – 3rd Patient Interview.

**Additional file 4.** Topic Guide – Physiotherapist Focus Groups.

## Data Availability

Not applicable.

## Abbreviations

CI: Chief investigator
COREQ: Consolidated criteria for reporting qualitative research
GCP: Good clinical practice
GDPR: General data protection regulation
ICF: International classification of function disability and health
IPA: Interpretative phenomenological analysis
NHS: National health service
PIS: Participant information sheet
PPI: Patient and public involvement
SMG: Study management group
SSG: Study steering group
TARN: Trauma and audit research network
