# Supplementary material for: Successful recovery following musculoskeletal trauma: protocol for a qualitative study of patients’ and physiotherapists’ perceptions
Source: BMC Musculoskelet Disord. 2021 Feb 10;22:163. doi: 10.1186/s12891-021-04035-9 (PMC7874566; doi:10.1186/s12891-021-04035-9)
Supplement: Supplementary file 1 — Additional file 1. Topic Guide – 1st Patient Interview. [file 12891_2021_4035_MOESM1_ESM.docx]

**Topic Guide – Patient Interviews**

| **Research Aims** | 1. To understand the patient journey following musculoskeletal trauma and whether perception of recovery changes through early to late stages of recovery  2. To explore patients’ views and perceptions on the definition of recovery and when they perceived to have achieved a successful recovery. | | |
| --- | --- | --- | --- |
| **Interview Section** | **Questions** | **Prompts** | **Aims** |
| Ethics & Consent | 1. Before we start, I want to check you are still happy and consent to participating in the interviews? 2. Just to remind you that the interview will be audio/video recorded and will be confidential. 3. Just to remind you that if any information is disclosed in today’s interview which would raise concerns for your immediate safety and well-being the researchers have a duty of care to disclose this information to relevant health care professionals in line with the Care Act 2014. 4. If you wish for to stop the interview at any point for a break, or stop the interview altogether, you are entitled to do this. If needed, we can break the interview into two sessions. 5. There are no right or wrong answers and I am interested in your own personal views, experiences and perceptions around your injury and your recovery. 6. You have the right not to answer a question if you do not want to. 7. Do you have any questions before we start? | - *Before we start, what are your level of symptoms right now?* - *Are you comfortable?* | - Check participant is happy to participate in the interviews still - Ensure the participant fully understands what the interview will involve and what is expected of them - Make sure the participant is comfortable and ready to begin. |
| Introductory Questions | 1. Can you tell me a bit about yourself? 2. Tell me a bit about how your accident/how you sustained your injury/ies 3. Can you tell me a bit more about your injuries in terms of what you have injured? 4. What treatment have you had since coming to hospital? 5. Can you tell me about what you can and can’t do compared to before your injury? | - *Age, occupation before Injury, hobbies before injury, family* - *Mechanism of Injury, can they remember the trauma taking place* - *Number of injuries, fractures, penetrating injuries, surgery?* - *Surgery, therapy input, pain team input* - *What can or can’t you do in terms of moving your limbs?* - *Does pain limit your movement?* - *How is your concentration?* - *Can you do day to day tasks e.g self care?* - *How is your mobility?* - *How about your family life and relationships?* - *How about thoughts about work?* | - To make the patient comfortable and at ease to be able to feel they can talk freely and openly in the interview - Build rapport with the participant - Gain an understanding of the participants life prior to the traumatic injury - Gain an understanding of the current function and injuries the participant has sustained |
| Transition Questions | 1. Can you tell me about your initial thoughts and feelings following your injury/injuries? 2. How do you feel about your injuries just now? | - *Thoughts about any physical changes or impairments?* - *Any feelings of worry, anxiety which you felt* - *Did you think about how your injury would affect you socially with friends and family?* - *Did you have any thoughts about your job?* | - Start to get the participant to think about their thoughts around their injury/recovery |
| Introduction to main questions | I am now going to focus on exploring your views around recovery and what you feel is successful recovery. I’m interested in what recovery means to you and your experience. | - *Do you have any questions?* - *If any of the questions don’t make sense please feel free to ask.* | - To introduce the main questions and topic for the interview. - To ensure that the participant knows they can ask if they don’t understand a question. |
| **Main Questions** | | | |
| Recovery | 1. Before this injury, what did the term recovery mean to you? | - *Have you had any previous injuries/surgeries when you had a recovery period? What did recovery mean to you at that time?* - *Can you tell me why these aspects were particularly important to you?* - *If no previous injuries – did recovery mean anything specific to you?* - *Can you tell me why these aspects were relevant to your definition of recovery?* | - Explore the definition of recovery prior to injury and why |
|  | 1. What does the term recovery mean to you now following this injury? | - *If your definition has changed, why?* - *Why are those particular aspects important when talking about recovery?* - *If your definition hasn’t changed, why do you think that is?* | - Explore whether definitions of recovery have changed following injury |
|  | 1. How has your recovery been so far? | - *Feelings towards any physical impairments?* - *Feelings towards day to day activities and mobility?* - *Feeling towards social aspects?* - *Work aspects?* - *Family and relationship aspects?* - *Is there any particular aspect which is most important to you?* | - Exploring attitudes towards recovery - What is most important to the participant at this moment in time. |
|  | 1. What do you think recovery will look like when you are discharged from hospital? | - *Why is this important to you?* | - Explore what they see as sufficiently recovered to be discharged |
|  | 1. Do you have any short term goals following your injury that you have thought of or that you want to achieve? | - *Do you anticipate any barriers to achieving these goals?* - *What do you think will help for you to achieve these goals?* | - To explore what is most important to the participant for recovery in the short term |
|  | 1. Looking to the future, do you have any long term goals following your injury which you have thought of or that you want to achieve? | - *Do you anticipate any barriers to achieving these long term goals?* - *What do you think will help to achieve these goals?* | - To explore what is important to the participant for recovery in the long term - To explore whether long term goals are important to the patient at this acute phase of injury. |
| Successful Recovery | 1. Can you tell me what a ‘successful recovery’ from these injuries would look like? | - *Are there any particular aspects which are more important to you?* - *Are there any physical/psychological/social/occupational aspects you have considered?* - *When do you think successful recovery will occur?* | - Explore what the participant understands and feels successful recovery is |
|  | 1. Do you anticipate any barriers to achieving a successful recovery? | - *If yes, what are these barriers?* - *Why do you think these will hinder you achieving a successful recovery?* - *If no, why do you think that?* | - To explore attitudes and potential environmental factors |
|  | 1. In contrast, do you anticipate anything which will help you achieving a successful recovery? | - *If yes, what are these?* - *Why do you think these will help achieve a successful recovery?* - *If no, why do you think that?* | - To explore attitudes and potential environmental factors |
|  | 1. From your experience so far, what are your impressions of what physiotherapists are looking for in terms of your recovery? | - *Why do you think this?* | - Gain an understanding of patient recovery so far as well as their experience of physiotherapists involved in their recovery |
| Final Questions & Summary | 1. Thank you for all your answers and comments. Is there anything else you would like to add before we end the interview? 2. The interview will now be transcribed and through a process called ‘member checking’ I can give you the opportunity to read through the transcript and add any further reflections you may have. At this stage, what are your thoughts about how you would like to receive the transcript? 3. The next interview will be in 6 months time – do you have any preference on how this interview will be conducted? | - *Any other comments that you would like to add around recovery?* - *Interviews can be conducted face to face at own home or University setting, or video call dependant on current COVID-19 government guidance at the time.* | - Finishing the interview - Informing participant what happens next. |
