# Supplementary material for: Successful recovery following musculoskeletal trauma: protocol for a qualitative study of patients’ and physiotherapists’ perceptions
Source: BMC Musculoskelet Disord. 2021 Feb 10;22:163. doi: 10.1186/s12891-021-04035-9 (PMC7874566; doi:10.1186/s12891-021-04035-9)
Supplement: Supplementary file 3 — Additional file 3. Topic Guide – 3rd Patient Interview. [file 12891_2021_4035_MOESM3_ESM.docx]

**Topic Guide – 3^rd^ Patient Interview**

| **Research Aims** | 1. To understand the patient journey following musculoskeletal trauma and whether perception of recovery changes through early to late stages of recovery  2. To explore patients’ views and perceptions on the definition of recovery and when they perceived to have achieved a successful recovery. | | |
| --- | --- | --- | --- |
| **Interview Section** | **Questions** | **Prompts** | **Aims** |
| Ethics & Consent | 1. Before we start, I want to check you are still happy and consent to participating in the interviews? 2. Just to remind you that the interview will be audio/video recorded and will be confidential. 3. Just to remind you that if any information is disclosed in today’s interview which would raise concerns for your immediate safety and well-being the researchers have a duty of care to disclose this information to relevant health care professionals in line with the Care Act 2014. 4. If you wish for to stop the interview at any point for a break, or stop the interview altogether, you are entitled to do this. 5. There are no right or wrong answers and I am interested in your own personal views, experiences and perceptions around your injury and your recovery. 6. You have the right not to answer a question if you do not want to. 7. Do you have any questions before we start? | - *Before we start, what are your level of symptoms right now?* - *Are you comfortable?* | - Check participant is happy to participate in the interviews still - Ensure the participant fully understands what the interview will involve and what is expected of them - Make sure the participant is comfortable and ready to begin. |
| Introductory Questions | 1. Can you give me an update on your recovery since our last interview? 2. Have you had any additional treatment since our last interview? 3. Can you tell me about what you can and can’t do compared to our last interview? | - *Have you made progress in your recovery?* - *Any setbacks in your recovery?* - *Surgery, therapy input, pain team input* - *What can or can’t you do in terms of moving your limbs?* - *Does pain limit your movement?* - *How is your concentration?* - *Can you do day to day tasks e.g self care?* - *How is your mobility?* - *How about your family life and relationships?* - *How about thoughts about work?* | - To make the patient comfortable and at ease to be able to feel they can talk freely and openly in the interview - Build rapport with the participant - Gain an understanding of the participants life since the last interview and any progress or setbacks in their recovery - Gain an understanding of the current function of the patient |
| Transition Questions | 1. How do you feel about your injuries just now? | - *Thoughts about any physical changes or impairments?* - *Any feelings of worry, anxiety?* - *Thoughts around your injury would affect you socially with friends and family?* - *Any thoughts about your job?* | - Start to get the participant to think about their thoughts around their injury/recovery |
| Introduction to main questions | I am now going to focus on exploring your views around recovery and what you feel is successful recovery. I’m interested in what recovery means to you and your experience and how this may or may not have changed since the first few weeks following your injury | - *Do you have any questions?* - *If any of the questions don’t make sense please feel free to ask.* | - To introduce the main questions and topic for the interview. - To ensure that the participant knows they can ask if they don’t understand a question. |
| **Main Questions** | | | |
| Recovery | 1. What does the term recovery mean to you now? | - *Why are those particular aspects important when talking about recovery?* - *Looking back from when you were first injured, do you feel the meaning of recovery has changed?* - *If so, why?* - *If it hasn’t changed, why do you think that is?* | - Explore whether definitions of recovery have changed following injury - Explore whether recovery perceptions have changed since the first interview? |
|  | 1. What are your feelings towards your recovery since our last interview 6 monts ago? | - *Feelings towards any physical impairments?* - *Feelings towards day to day activities and mobility?* - *Feeling towards social aspects?* - *Work aspects?* - *Family and relationship aspects?* - *Is there any particular aspect which is most important to you?* | - Exploring attitudes towards recovery - What is most important to the participant at this moment in time. |
|  | 1. Do you have any short term goals following your injury that you have thought of or that you want to achieve currently? | - *Do you anticipate any barriers to achieving these goals?* - *What do you think will help for you to achieve these goals?* | - To explore what is most important to the participant for recovery in the short term |
|  | 1. Looking to the future, do you have any long term goals following your injury which you have thought of or that you want to achieve? | - *Do you anticipate any barriers to achieving these long term goals?* - *What do you think will help to achieve these goals?* | - To explore what is important to the participant for recovery in the long term - To explore whether long term goals are important to the patient at this acute phase of injury. |
| Successful Recovery | 1. Can you tell me what a ‘successful recovery’ from your injury looks like now 12 months following the injury? | - *Are there any particular aspects which are more important to you?* - *Are there any physical/psychological/social/occupational aspects you have considered?* - *When do you think successful recovery will occur?* - *Do you think your opinion has changed on what successful recovery is since the last interview?* - *If so why?* - *If not, why?* | - Explore what the participant understands and feels successful recovery is |
|  | 1. Do you feel you are ‘fully recovered’? | - *If yes, why?* - *If not, why?* - *What do you feel you will need to achieve to be fully recovered?* - *How long do you anticipate full recovery will take?* | - Explore definitions of successful recovery at 12 months |
|  | 1. In terms of achieving your goals and successful recovery, do you anticipate any barriers to achieving a successful recovery? | - *If yes, what are these barriers?* - *Why do you think these will hinder you achieving a successful recovery?* - *If no, why do you think that?* | - To explore attitudes and potential environmental factors |
|  | 1. In contrast, do you anticipate anything which will help you achieving a successful recovery or your goals? | - *If yes, what are these?* - *Why do you think these will help achieve a successful recovery?* - *If no, why do you think that?* | - To explore attitudes and potential environmental factors |
|  | 1. From your experience over the last 12 months, what are your impressions of what physiotherapists are looking for in terms of your recovery? | - *Why do you think this?* - *Are there any differences which you have noticed from when you were in hospital?* | - Gain an understanding of patient recovery so far as well as their experience of physiotherapists involved in their recovery |
| Final Questions & Summary | 1. Thank you for all your answers and comments. Is there anything else you would like to add before we end the interview? 2. The interview will now be transcribed and through a process called ‘member checking’ I can give you the opportunity to read through the transcript and add any further reflections you may have. At this stage, what are your thoughts about how you would like to receive the transcript? 3. Thank you for participating in the study, there are no further interviews | - *Any other comments that you would like to add around recovery?* | - Finishing the interview - Informing participant what happens next. |
