# Supplementary material for: Successful recovery following musculoskeletal trauma: protocol for a qualitative study of patients’ and physiotherapists’ perceptions
Source: BMC Musculoskelet Disord. 2021 Feb 10;22:163. doi: 10.1186/s12891-021-04035-9 (PMC7874566; doi:10.1186/s12891-021-04035-9)
Supplement: Supplementary file 4 — Additional file 4. Topic Guide – Physiotherapist Focus Groups. [file 12891_2021_4035_MOESM4_ESM.docx]

**Topic Guide – Physiotherapist Focus Groups**

| **Research Aims** | 1. To explore the physiotherapists’ perceptions of what they define as a successful patient recovery. 2. To explore views and perceptions of physiotherapists regarding outcome measures that are useful to assess recovery. | | |
| --- | --- | --- | --- |
| **Interview Section** | **Questions** | **Prompts** | **Aims** |
| Ethics | 1. Before we start, I would like to thank you all for participating in this focus group. Just to remind you that the interview will be audio/video recorded and the recordings/transcripts will remain confidential at all times. 2. We would like to emphasise there are no right or wrong answers and every opinion matters, and we are interested in your views and personal experiences and we welcome all opinions. We would like to remind you that all discussions within this focus group are confidential in order to encourage everyone to discuss and talk freely about the topic. 3. Do you have any questions before we start? | - *Can I confirm that everyone has read the participant information sheet and signed the consent form?* | - Introduce the focus groups and ensure all participants are informed of how the group will run. - Ensure that all participants are comfortable in discussing their views and to manage any potential power imbalances within the group |
| Introductory Question | 1. Let’s start by introducing ourselves and your role and how long you have you been qualified | - *Age/gender/ethnicity* - *Role – senior/junior* - *How many years been qualified?* - *How long have you worked specifically with trauma patients?* - *Does anyone specialise in musculoskeletal trauma or do these patients form part of your caseload?* | - Encourage participants start to engage and talk within the group and become comfortable with other participants |
| Transition Questions | 1. What do you define as musculoskeletal trauma? 2. Does anyone have any thoughts around how well people recover from a traumatic injury? | - *Any particular mechanism or injuries which come to mind?* - *Which structures could be involved?* - *Why do you think people recover well or not recover as well?* | - To set the context of the type of patients which is relevant to this study - Start to introduce thinking around recovery in this population |
| Introduction to main questions | We are now going to move on to the main questions for the focus group. For the purpose of this focus group we define musculoskeletal trauma as any musculoskeletal structure which has been damaged due to a traumatic event.  We are interested in your own views and opinions around recovery following musculoskeletal trauma, and particularly on how you define successful recovery and outcome measures you find useful to assess recovery. | - *Does anyone have any questions?* - *If any question doesn’t make sense please feel free to ask* | - To review the main aims of the study |
| Main Questions | 1. What do you understand by the term recovery? | - *Why do you think these aspects are important?* - *Is there one particular aspect which stands out?* - *Do you think recovery is individual?* | - Generate discussion around recovery and what the views are on a definition of recovery |
|  | 1. What do you understand by the term outcome? | - *Do you feel recovery and outcome are different?* - *Can you elaborate further?* |  |
|  | 1. Can you tell me what you think would characterise a successful recovery following musculoskeletal trauma? | - *Why do you think this?* - *How do you think that maybe influenced by stages of recovery?* - *Do you think successful recovery differs depending on severity of injury?* - *Do you think successful recovery is individual to the patient?* | - Gain an understanding of the definition of successful recovery - Generate discussion around the definition of successful recovery |
|  | 1. From your own experience, what are your impressions of what patients are looking for in terms of recovery? | - *Why do you think this?* - *Do you think this changes over time?* | - Gain an understanding of physiotherapists perception of what is important to patients in terms of recovery |
|  | 1. What do you think is important to patients to achieve a successful recovery | - *Are there any physical aspects to consider?* - *What about any psychological aspects when considering successful recovery?* - *What about family or social interaction?* - *Do you think returning to work is important to patients?* | - Gain an understanding of what they feel is important to the patient - Does this align to the definition of successful recovery from a physiotherapist |
|  | 1. When would you discharge a patient from a therapy point of view following a traumatic injury? | - *What are the criteria you would use for discharge from a therapy point of view?* - *Any functional criteria?* - *Any psychological criteria?* - *Any physical criteria?* - *Is this related to recovery?* - *Do you think patient goals are important?* - *Are there any particular outcomes which you look for before discharging?* - *Do you focus on successful recovery for discharge?* | - Exploring whether discharge is related to recovery or successful recovery definitions |
|  | 1. What outcome measures do you think are useful in measuring a patient’s recovery? | - *Any particular outcome measures you use regularly?* - *Are these PROMS or PBOM?* - *If not, why don’t you use outcome measures?* - *How do you evaluate recovery and outcome in musculoskeletal trauma?* - *Within musculoskeletal trauma what would be essential to evaluate?* - *Evaluate from patient perspective or performance based.* | - To understand what outcome measures are being used in practice - To understand what physiotherapists, feel are the most important aspects to measure recovery in a outcome measure |
| Final Questions & Summary | 1. Thank you for all your comments and discussion during the focus group. 2. Does anyone like to add anything further before we end the focus group? 3. The focus group will now be transcribed but all information will be kept confidential and you will not be identifiable from the transcripts. | - *Any questions?* | - Concluding the interview - Informing of next steps |
